# Supplementary material for: Dielectric Barrier Discharge Plasma Jet (DBDjet) Processed Reduced Graphene Oxide/Polypyrrole/Chitosan Nanocomposite Supercapacitors
Source: Polymers (Basel). 2021 Oct 18;13(20):3585. doi: 10.3390/polym13203585 (PMC8538378; doi:10.3390/polym13203585)
Supplement: Supplementary file 1 [file polymers-13-03585-s001.zip › polymers-1385483-supplementary.pdf]

(Supplementary Information)

Dielectric barrier discharge plasma jet (DBDjet) processed reduced graphene oxide/polypyrrole/chitosan nanocomposite supercapacitors

Chen Liu[1,2], Cheng-Wei Hung[3], I-Chung Cheng[3], Cheng-Che Hsu[4], I-Chun Cheng[5,6,7], Jian-Zhang Chen[1,2,7]

[1] Graduate Institute of Applied Mechanics, National Taiwan University, Taipei City 10617, Taiwan

[2] Advanced Research Center for Green Materials Science and Technology, National Taiwan University, Taipei City 10617, Taiwan

[3] Department of Mechanical Engineering, National Taiwan University, Taipei City 10617, Taiwan

[4] Department of Chemical Engineering, National Taiwan University, Taipei City 10617, Taiwan

[5] Graduate Institute of Photonics and Optoelectronics, National Taiwan University, Taipei City 10617, Taiwan

[6] Department of Electrical Engineering, National Taiwan University, Taipei City 10617, Taiwan

[7] Innovative Photonics Advanced Research Center (i-PARC), National Taiwan University, Taipei City 10617, Taiwan

* Correspondence: TEL: +886-2-33665694; Email: jchen@ntu.edu.tw (J.Z.C.)

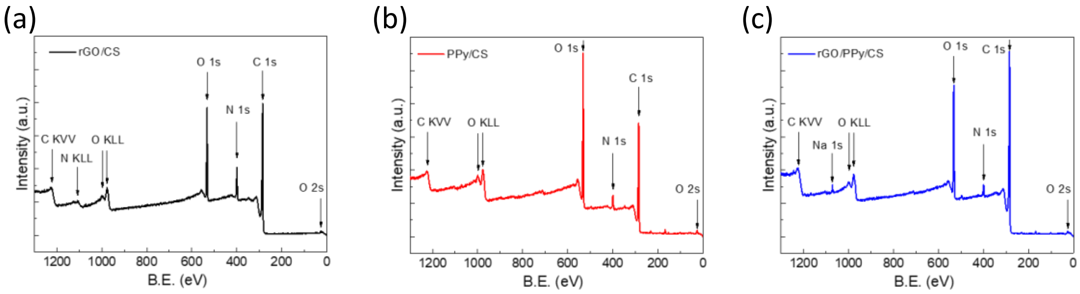

Figure S1. XPS analysis of electrodes for (a) rGO/CS, (b) PPy/CS, and (c) rGO/PPy/CS pastes on carbon cloth.

Table S1. Atomic ratios of C, O, N, Na, S, and Si as calculated from XPS spectra.

	C1s	O1s	N1s	Na1s	S2p	Si2p
rGO/CS	72.9%	17.0%	9.7%	0.4%	0.0%	0.0%
PPy/CS	63.7%	28.3%	5.3%	0.6%	1.1%	1.0%
rGO/PPy/CS	76.1%	18.2%	3.5%	0.7%	0.7%	0.9%

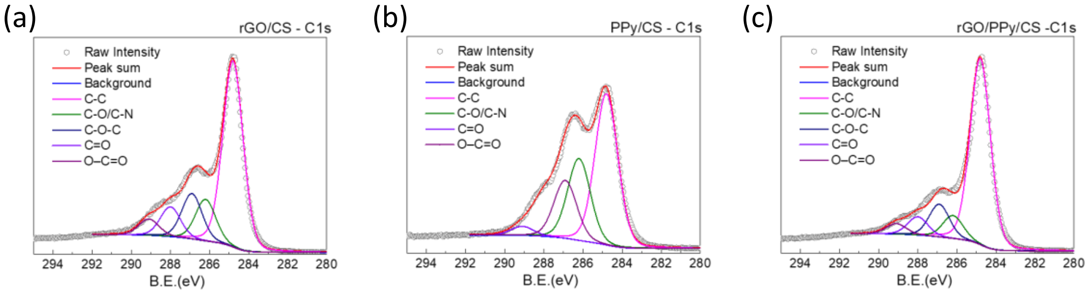

Figure S2. XPS spectra of C1s for (a) rGO/CS, (b) PPy/CS, and (c) rGO/PPy/CS paste on carbon cloth.

Table S2. XPS C1s spectra of electrode with rGO/CS, PPy/CS and rGO/PPy/CS pastes on carbon cloth

Bonding states/ Binding energy(eV)	C-C (284.8)	C-O/C-N (286.2)	C-O-C (286.9)	C=O (288)	O–C=O (289.2)
rGO/CS	58.4%	13.0%	14.2%	9.3%	5.1%
PPy/CS	45.1%	24.4%	17.3%	10.5%	2.7%
rGO/PPy/CS	68.9%	8.5%	12.0%	6.7%	3.9%
Reference	[1]	[1]	[2]	[1]	[3]

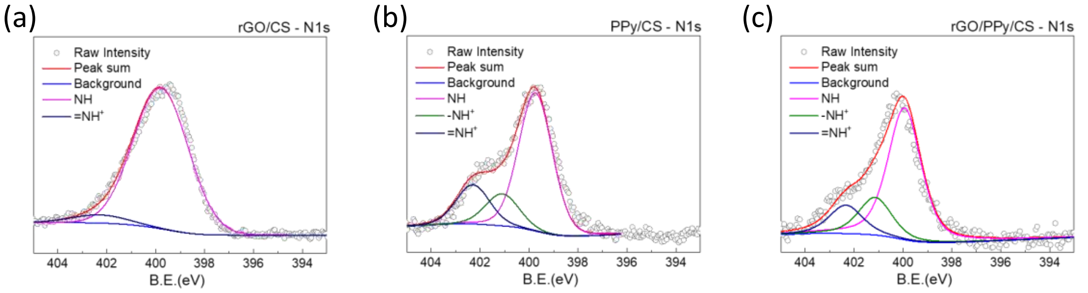

Figure S3. XPS spectra of N1s for (a) rGO/CS, (b) PPy/CS, and (c) rGO/PPy/CS paste on carbon cloth.

Table S3. XPS N1s spectra of electrode with rGO/CS, PPy/CS and rGO/PPy/CS pastes on carbon cloth

Bonding states/ Binding energy (eV)	$–NH–$ (399.8)	$–NH^+–$ (401.1)	$= NH^+–$ (402.3)
rGO/CS	94.5%	0.0%	5.5%
PPy/CS	66.0%	15.3%	18.7%

rGO/PPy/CS	66.6%	19.0%	14.4%
Reference	[4]	[4]	[4]

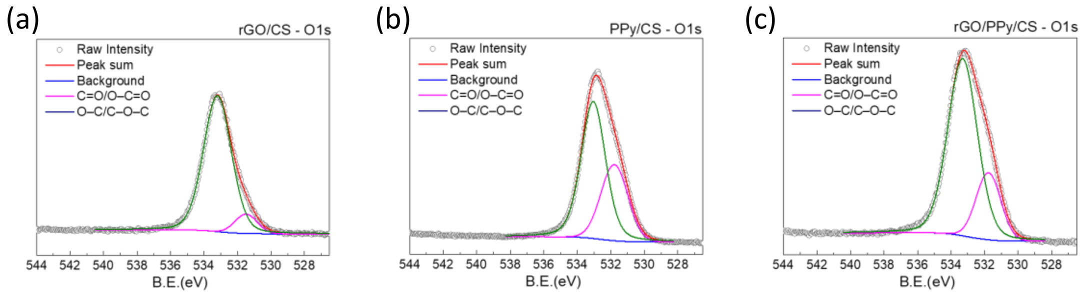

Figure S4. XPS spectra of O1s for (a) rGO/CS, (b) PPy/CS, and (c) rGO/PPy/CS pastes on carbon cloth.

Table S4. XPS O1s spectra of electrode with rGO/CS, PPy/CS, and rGO/PPy/CS pastes on carbon cloth

Bonding states/ Binding energy (eV)	C=O / O–C=O (531.8)	O–C / C–O–C (533.1)
rGO/CS	9.4%	90.6%
PPy/CS	35.1%	64.9%
rGO/PPy/CS	22.4%	77.6%
reference	[4]	[4]

Table S5. Comparison of areal capacitance of SCs with different materials listed in literatures.

Material	Electrolyte	Areal capacitance	Year	Ref.
rGO/PPy/CS	1 M H_2SO_4/PVA gel electrolyte	72.79 mF/cm^2 @ 0.333 mA/cm^2	2021	this study
PEDOT/rGO/CS	1 M H_2SO_4/PVA gel electrolyte	399.33 mF/cm^2 @ 3.333 mA/cm^2	2021	[5]
NNA/PPy	0.5 M Na_2SO_4 aqueous electrolyte	376.90 mF/cm^2 @ 1 mV/s	2015	[6]
rGO/PANI/CS	1 M H_2SO_4/PVA gel electrolyte	195.83 mF/cm^2 @ 0.333 mA/cm^2	2020	[7]
rGO/SnO_2	1 M H_2SO_4/PVA gel electrolyte	34.73 mF/cm^2 @ 0.083 mA/cm^2	2021	[8]

PPy/GO	1 M KCl/filter paper solid-state electrolyte	22.80 mF/cm^2 @ 0.1 mA/cm^2	2014	[9]
rGO	1 M H_2SO_4/PVA gel electrolyte	22.43 mF/cm^2 @ 0.083 mA/cm^2	2019	[10]
e-WO_3/PPy	H_2SO_4/PVA solid-state electrolyte	11.38 mF/cm^2 @ 20 mV/s	2015	[11]
GO/rGO	1 M $TEABF_4$ liquid electrolyte	~2.70 mF/cm^2 @ 20 mV/s	2011	[12]

Note. NNC: Ni nanocones.

References

1. Parnell, C.M.; Chhetri, B.P.; Mitchell, T.B.; Watanabe, F.; Kannarpady, G.; RanguMagar, A.B.; Zhou, H.; Alghazali, K.M.; Biris, A.S.; Ghosh, A. simultaneous electrochemical Deposition of Cobalt Complex and poly (pyrrole) thin Films for supercapacitor electrodes. *Scientific Reports* **2019**, *9*, 1-13.
2. Zhang, L.; Li, Y.; Zhang, L.; Li, D.-W.; Karpuzov, D.; Long, Y.-T. Electrocatalytic oxidation of NADH on graphene oxide and reduced graphene oxide modified screen-printed electrode. *International Journal of Electrochemical Science* **2011**, *6*, 819-829.
3. Sun, K.G.; Chung, J.S.; Hur, S.H. Durability improvement of Pt/RGO catalysts for PEMFC by low-temperature self-catalyzed reduction. *Nanoscale research letters* **2015**, *10*, 1-7.
4. Bashid, H.A.A.; Lim, H.N.; Kamaruzaman, S.; Rashid, S.A.; Yunus, R.; Huang, N.M.; Yin, C.Y.; Rahman, M.M.; Altarawneh, M.; Jiang, Z.T. Electrodeposition of polypyrrole and reduced graphene oxide onto carbon bundle fibre as electrode for supercapacitor. *Nanoscale research letters* **2017**, *12*, 1-10.
5. Tseng, C.-H.; Lin, H.-H.; Hung, C.-W.; Cheng, I.-C.; Luo, S.-C.; Cheng, I.-C.; Chen, J.-Z. Electropolymerized Poly (3, 4-ethylenedioxythiophene)/Screen-Printed Reduced Graphene Oxide–Chitosan Bilayer Electrodes for Flexible Supercapacitors. *ACS Omega* **2021**.
6. Xu, C.; Li, Z.; Yang, C.; Zou, P.; Xie, B.; Lin, Z.; Zhang, Z.; Li, B.; Kang, F.; Wong, C.P. An ultralong, highly oriented nickel-nanowire-array electrode scaffold for high-performance compressible pseudocapacitors. *Advanced Materials* **2016**, *28*, 4105-4110.
7. Tseng, C.-H.; Hsin, J.-C.; Tsai, J.-H.; Chen, J.-Z. Dielectric-barrier-discharge jet

treated flexible supercapacitors with carbon cloth current collectors of long-lasting hydrophilicity. *Journal of the Electrochemical Society* **2020**, *167*, 116511.

8. Chang, J.-H.; Chen, S.-Y.; Kuo, Y.-L.; Yang, C.-R.; Chen, J.-Z. Carbon Dioxide Tornado-Type Atmospheric-Pressure-Plasma-Jet-Processed rGO-SnO2 Nanocomposites for Symmetric Supercapacitors. *Materials* **2021**, *14*, 2777.
9. Zhou, H.; Han, G.; Xiao, Y.; Chang, Y.; Zhai, H.-J. Facile preparation of polypyrrole/graphene oxide nanocomposites with large areal capacitance using electrochemical codeposition for supercapacitors. *Journal of Power Sources* **2014**, *263*, 259-267.
10. Fan, C.-F.; Chien, Y.-C.; Hsu, C.-C.; Cheng, I.-C.; Chien, L.-H.; Chen, J.-Z. Flexible reduced graphene oxide supercapacitors processed using atmospheric-pressure plasma jet under various temperatures adjusted by flow rate and jet-substrate distance. *MAterials Research Express* **2019**, *7*, 015602.
11. Zhu, M.; Huang, Y.; Huang, Y.; Meng, W.; Gong, Q.; Li, G.; Zhi, C. An electrochromic supercapacitor and its hybrid derivatives: quantifiably determining their electrical energy storage by an optical measurement. *Journal of Materials Chemistry A* **2015**, *3*, 21321-21327.
12. Gao, W.; Singh, N.; Song, L.; Liu, Z.; Reddy, A.L.M.; Ci, L.; Vajtai, R.; Zhang, Q.; Wei, B.; Ajayan, P.M. Direct laser writing of micro-supercapacitors on hydrated graphite oxide films. *Nature Nanotechnology* **2011**, *6*, 496-500.
